# Steroid resistance and concomitant respiratory infections: A challenging battle in pulmonary clinic

**DOI:** 10.17179/excli2017-425

**Published:** 2017-06-29

**Authors:** Kamal Dua, Nicole G. Hansbro, Philip M. Hansbro

**Affiliations:** 1 Discipline of Pharmacy, Graduate School of Health, University of Technology,Sydney, Ultimo NSW 2007, Australia; 2 School of Biomedical Sciences and Pharmacy, The University of Newcastle, Callaghan, NSW2308, Australia; 3 Priority Research Centre for Healthy Lungs, Hunter Medical Research Institute, J Lot 1 Kookaburra Circuit, New Lambton Heights, Newcastle, NSW 2305, Australia

## ⁯

Dear Editor,

Globally, the viral respiratory infections are one of the major health problems. The investigations in this area are becoming more challenging because of the complexity of relationship between the host's defences and microbial virulence (Saturni et al., 2015[28]). Particularly, the role of infections in asthma can cause wheezing as an “inducer” and can also act as a “protector” against allergic airway disease (Busse et al., 2010[4]). According to the “hygiene” hypothesis, early life infection may protect against asthma. If the parents of the offspring have asthma or allergies, the chances of exacerbations with viral respiratory infections particularly provoke wheezing in the early life and lead to the development of asthma in later stages (Budden et al., 2017[3]; Okada et al., 2010[26]). Sigurs et al. (2000[29]) have shown that the family history of asthma along with severe respiratory syncytial virus (RSV) infections increases the development of asthma in children at the age of seven. Nowadays, increasing number of asthma patients with steroid-resistance and coexisting respiratory viral infections has severely affected the cost of treating asthma patients (Durham et al., 2011[10]). Moreover, viral respiratory infections are also one of the major causes of exacerbations further deteriorating the quality of life for these patients.

Various studies have provided mechanistic insights showing an association of respiratory viral (RSV, rhinovirus) and bacterial (Chlamydia, Mycoplasma) infections with asthma (Hansbro et al., 2014[12]). Using the mouse model, it has been well demonstrated that chlamydia respiratory infections during early life modify lung physiology where it increases the severity of allergic airway disease by targeting factor like interleukin-13 (IL-13) (Starkey et al., 2013[30]) and tumor necrosis factor-related apoptosis-inducing ligand (TRAIL) (Starkey et al., 2014[32][31]). Various other factors that have been investigated to play a role in the susceptibility to respiratory viral infections in allergic airway diseases include monocyte chemoattractant protein-1 (MCP-1), keratinocyte-derived protein chemokine (KC) (Nguyen et al., 2016[24]) and receptor for advanced glycation end-products (RAGE) (Arikkatt et al., 2017[1]). 

It has been demonstrated in mice models that IL-13 impaired antiviral immune responses in various respiratory diseases including asthma and chronic obstructive pulmonary disease (COPD). These impaired responses predisposed these mice to severe influenza infection that exacerbated the underlying disease via increased expression of micro-RNA-21 (miRNA-21) and phosphoinositide 3-kinase (PI3K). This indicates the potential of PI3K inhibitors, anti-IL-13 and miRNA-21 antagomirs as novel therapeutic interventions in management of allergic airways diseases (Dua et al., 2017[9]; Starkey et al., 2014[32][31]). Furthermore, miR-21/PI3K/histone deacetylase (HDAC) 2 axis has recently been reported to drive severe, steroid-insensitive experimental asthma (Kim et al., 2017[15]). In a dual T-helper 2/T-helper 17 (Th2/Th17) model of steroid-resistant asthma, IL-13-mediated and signal transducer and activator of transcription 6 (STAT6)-dependent airway hyper-responsiveness (AHR) and mucus metaplasia was observed, however, IL-13 was not identified to be directly contributing to airway/tissue inflammation. Similarly, in the same mixed model, interleukin-17A (IL-17A) was identified as an independent contributor to AHR with only partial mediation of inflammation and mucus metaplasia (Manni et al., 2016[19]). 

Specifically with PI3K, increased PI3K catalytic subunit p110α (PI3K-p110α0 activity has been demonstrated to increase susceptibility of individuals with COPD to influenza infections (Chen-Yu Hsu et al., 2015[7]). This was evident with the increased viral entry and replication (increased viral titre) in COPD primary bronchial epithelial cells (pBECs) and pulmonary inflammation along with compromised lung function in infected mice with experimental COPD (Beckett et al., 2013[2]; Chen-Yu Hsu et al., 2015[7]). Long et al. (2016[18]) have shown the involvement of natural killer (NK) cells in persistent airway inflammation and AHR during later stages of RSV infection in mice, where targeting NK cells therapeutically may be a novel approach to improve recurrent wheezing following to RSV infection.

One of the recent studies emphasized on the involvement of bromodomain and extra terminal (BET) proteins in regulation of AHR and airway inflammation in interferon-γ (IFNγ)/lipopolysaccharide (LPS, an endotoxin) and RSV-induced steroid-resistant exacerbations models. They presented the therapeutic potential of BET inhibitor in suppressing macrophage-driven steroid-resistant exacerbations (Nguyen et al., 2016[23]). In COPD, combination of roflumilast N-oxide and dexamethasone was demonstrated to produce additive anti-inflammatory effects in COPD pBECs by increasing the expression of mitogen-activated protein kinase phosphatase 1 (MKP1; also known as dual specificity protein phosphatase 1, (DUSP1) and enhancing inhibitory effects on phospho-p38 and nuclear factor-κB (NFκB) (Milara et al., 2015[21]). 

The investigation of Chambers et al. (2015[6]) into identifying immunological differences between steroid-sensitive and steroid-resistant asthma demonstrated patients with steroid resistance asthma to produce significantly high levels of IL-17A and IFN-γ. Calcitriol treatment in both an *in-vitro* (peripheral blood mononuclear cell, PBMCs) and *in-vivo* (steroid resistance asthma patients) settings demonstrated an improvement in clinical response to oral glucocorticoids probably by directing the cytokine profile of steroid-resistance asthma patients towards the steroid-sensitive immune phenotype. With an aim of increasing patient compliance in COPD and steroid-refractory asthma, Onions and his co-workers designed and optimized various chemical compounds that could produce sustained action post-inhalation (Onions et al., 2016[27]). Further, microRNA-9 (miRNA-9) was investigated as another potential therapeutic target by Li et al. (2015[17]), where it was hypothesised to regulate glucocorticoid receptor (GR) signalling and steroid-resistant AHR in steroid-resistant asthma.

Tian et al. (2016[33]) emphasised on the apoptosis of inflammatory cells which is an important prerequisite feature in clearing airway inflammation induced by insults such as allergens. They demonstrated the potential of Bcl-2 inhibitors ABT-737 or ABT-199 as promising therapeutic tools in the treatment of corticosteroid-insensitive neutrophilic airway inflammation (Tian et al., 2016[33]). Another potential therapeutic intervention included an anti-RSV neutralizing antibody (palivizumab) which has recently been approved for the prevention of severe RSV infection in high-risk patients. This antibody was tested in mice model where the antibody was administered once either (a) 24 hours prior to infection as prophylaxis or (b) 48 hours post-infection (inoculation with RSV). They showed attenuated RSV replication in the lower respiratory tract as well as significant reduction in the cytopathic effect of virus particularly in the respiratory epithelial cells and in the immune response elicited by RSV in response to the treatment (Carbonell-Estrany and Quero, 2002[5]; Group, 1998[11]; Mejías et al., 2004[20]). 

Hines and colleagues investigated molecular processes involved in structural remodelling as a consequence of repeated respiratory viral infections during early childhood. They demonstrated distinct responses from the macrophages and mast cells along with abnormal re-epithelization resulting in various structural defects using Sendai virus infection model in weanling rats (an atopic asthma susceptible strain, Brown Norway, and a non-atopic asthma resistant strain, Fischer 344) (Hines et al., 2014[14]). A translational investigation using a blend of genetic animal model and *in-vitro* human studies identified an innate immunity scavenger receptor MARCO gene to be associated with increased susceptibility of children to RSV infection (High et al., 2016[13]). Also a clinical trial investigating the efficacy and safety of long-term treatment with anti-IgE antibody, omalizumab, in children with uncontrolled severe allergic asthma demonstrated it to be well tolerated with improvements in asthma control (Odajima et al., 2017[25]). 

A multicenter, randomized, double-blind, placebo-controlled, parallel-group study assessed the safety and efficacy of inhaled Zanamivir in preventing infection in adult and adolescent subjects susceptible to influenza infection particularly against the circulating strains of the 2000-2001 influenza season in the Northern Hemisphere (influenza A/New Calendonia/20/99-1ike and influenza B/ Sichuan/379/99-like). Zanamivir was demonstrated to be well-tolerated with a placebo comparable safety profile (LaForce et al., 2007[16]). Likewise, a randomized, double-blind, placebo-controlled, crossover phase 1 study evaluated the safety of an inhaled antiviral DAS181 (Fludase®) in adult subjects with well-controlled asthma (Colombo et al., 2016[8]; Zenilman et al., 2015[34]) which was a part of clinical trial where DAS181 was shown to reduce viral load (Moss et al., 2012[22]). 

Though, there are number of translational and clinical studies performed worldwide to investigate molecular mechanisms interlinking influenza infection and allergic airway diseases along with the ongoing search for potential therapeutic interventions, there are still many questions that remain unaddressed. Some of these impediments include patterns of inflammation involved due to various respiratory viruses and multiple genes and their products, which underpin the regulatory mechanisms driving the disease pathology. 

## Notes

Kamal Dua and Philip M. Hansbro (Priority Research Centre for Healthy Lungs, Hunter Medical Research Institute, J Lot 1 Kookaburra Circuit, New Lambton Heights, Newcastle, NSW 2305, Australia; Tel: +61 2 4042 20203, Fax: +61 2 4042 0024, e-mail: philip.hansbro@newcastle.edu.au) contributed equally as corresponding authors.
